# Supplementary material for: Utilising an in silico model to predict outcomes in senescence-driven acute liver injury
Source: NPJ Regen Med. 2024 Sep 30;9:26. doi: 10.1038/s41536-024-00371-1 (PMC11442582; doi:10.1038/s41536-024-00371-1)
Supplement: Supplementary file 1 — Supplementary Information [file 41536_2024_371_MOESM1_ESM.pdf]

Supplementary Material for: **Utilising an in silico model to predict outcomes in senescence-driven acute liver injury**

Authors

Candice Ashmore-Harris[1†], Evangelia Antonopoulou[2†], Rhona E. Aird[1], Tak Yung Man[1], Simon M. Finney[2], Annelijn M. Speel[1], Wei-Yu Lu[3], Stuart J. Forbes[1], Victoria L. Gadd[1# A], Sarah L. Waters[2# B]

[†]These authors contributed equally to this work and share first authorship
[#]These authors contributed equally to this work and share senior authorship

1. Centre for Regenerative Medicine, Institute for Regeneration and Repair, University of Edinburgh, Edinburgh BioQuarter, 5 Little France Drive, Edinburgh, EH16 4UU, UK.
2. Mathematical Institute, University of Oxford, Andrew Wiles Building, Radcliffe Observatory Quarter (550), Woodstock Road, Oxford, OX2 6GG, UK
3. Centre for Inflammation Research, Institute for Regeneration & Repair, University of Edinburgh, Edinburgh BioQuarter, 4-5 Little France Drive, Edinburgh, EH16 4UU, UK.

Correspondence:

[A] Corresponding author – experimental (victoria.gadd@ed.ac.uk)

[B] Corresponding author – mathematical (waters@maths.ox.ac.uk)

Contents

	Page number
Supplementary Figure 1	3
Supplementary Figure 2	4
Supplementary Figure 3	5
Supplementary Figure 4	6
Supplementary Figure 5	7
Supplementary Figure 6	9
Supplementary Figure 7	10
Supplementary Table 1	11
Supplementary Table 2	12
Supplementary Note 1 – Steady states & Linear stability analysis	13
Supplementary Note 2	15
Supplementary Note 3	17

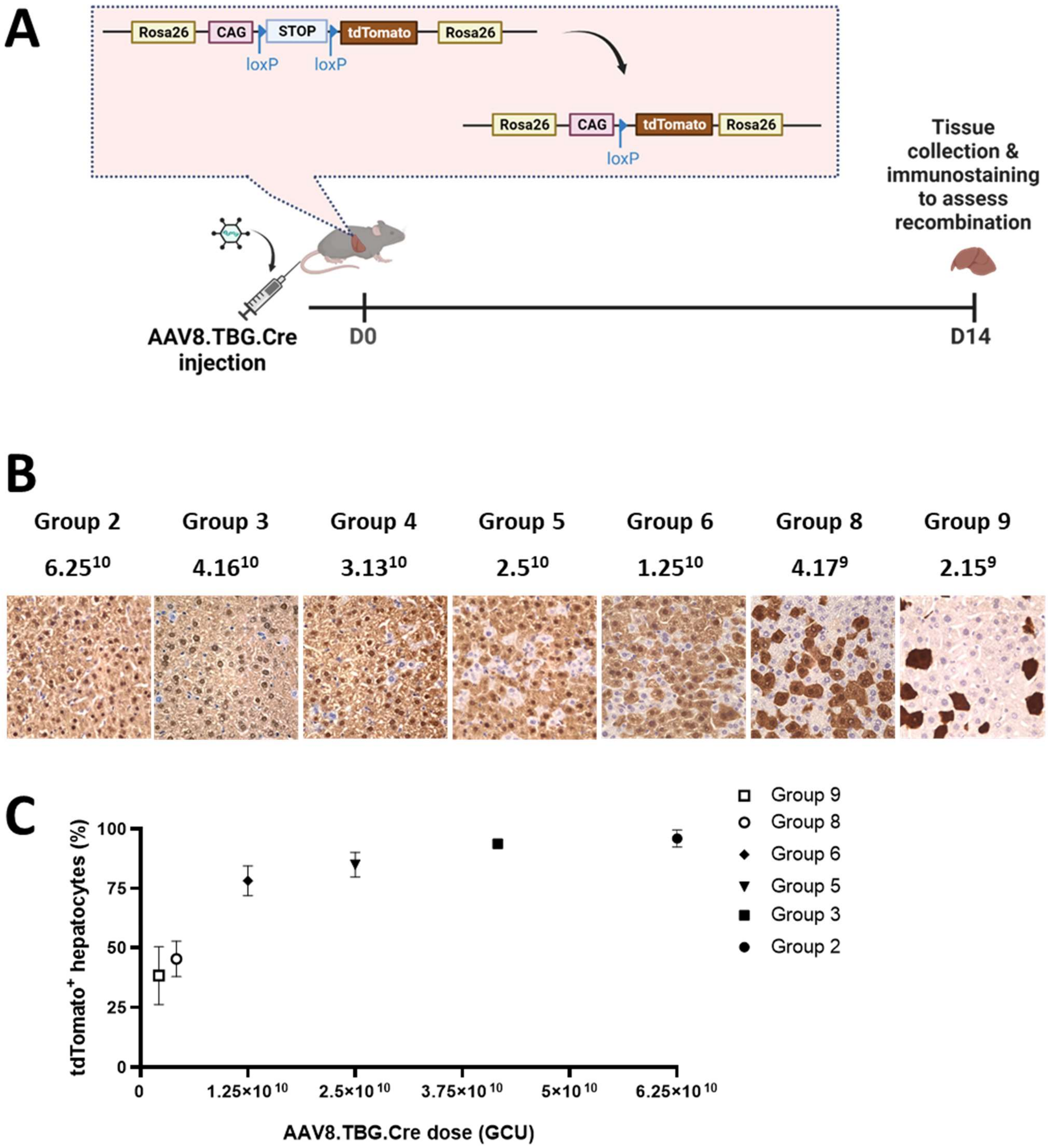

Supplementary Figure 1- Rosa26$^{LSL\text{-}TdTomato}$ AAV8-TBG-Cre dose response to assess recombination efficiency. **a)-** Graphical schematic for the recombination dose response study. Inset demonstrates tissue specific Cre recombinase expression mediated induction of tdTomato expression in the Rosa26$^{LSL\text{-}TdTomato}$ mouse model. Due to the hepatotropic nature of the AAV8.TBG.Cre, the dose injected impacts the proportion of hepatocytes that express tdTomato as a result of Cre-mediated recombination, allowing assessment of recombination efficiency with different doses based on quantification of the proportion of hepatocytes that are tdTomato positive. Elements of this panel were produced using biorender.com. **b)-** Representative image panels from histological sections immunostained for tdTomato demonstrating the changing proportion of positively stained cells. Labels correspond to the equivalent Group in the Mdm2 study (top) and the AAV8 dose in GCU (bottom). **C)-** Quantification of recombination demonstrating the mean proportion of recombined hepatocytes for each dose as % tdTomato positive cells (N=3 mice per group, N≥5 FOVs analysed per mouse, error bars ±SEM).

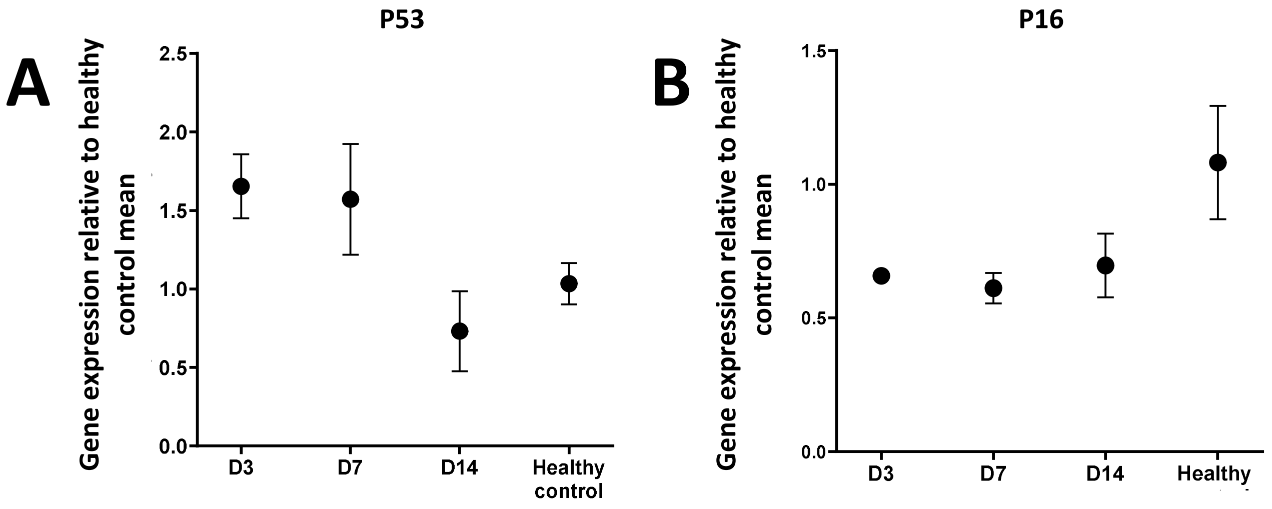

Supplementary Figure 2- Expression of senescence markers in whole liver tissue of Mdm2 mice administered an AAV8.TBG.Cre dose commensurate to a moderate dose. Expression of **a)** p53, b) p16 was analysed at the indicated time points. qPCR results were normalised to PPIA housekeeper and expressed as fold change relative to healthy control mean expression. N=3-5 mice per timepoint analysed. Error bars are SEM. Ordinary One-Way ANOVA relative to healthy control mean with Dunnett’s Multiple Comparisons test.

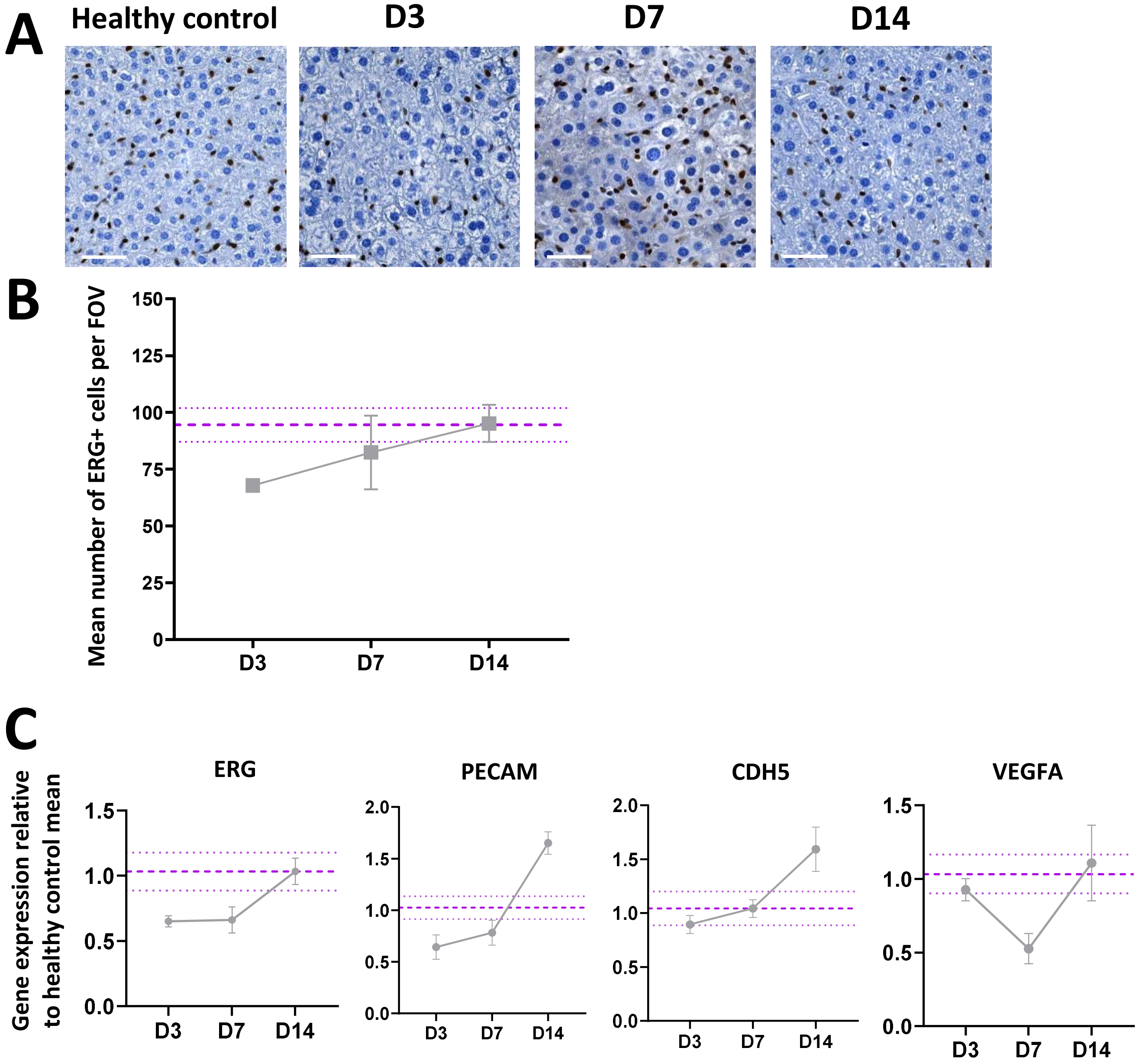

Supplementary Figure 3- Tissue and gene expression of endothelial markers in whole liver tissue of Mdm2 mice administered an AAV8.TBG.Cre dose commensurate to a moderate dose.

a) Representative histological micrographs of ERG (endothelial nuclear cell marker) at D3, D7 and D14 following induction of moderate senescence injury induction. Scale bars are 50µm.

b) Quantification of immunostaining in [A] as number of positively stained nuclei per FOV. ≥10 FOVs quantified per animal. N=3-5 animals per timepoint. Error bars are ±SEM.

c) mRNA expression of the endothelial markers ERG, PECAM, CDH5 and VEGFA was analysed at indicated time points. qPCR results were normalised to PPIA housekeeper and expressed as fold change relative to healthy control mean expression. N=3-5 mice per timepoint analysed. Error bars are ±SEM.

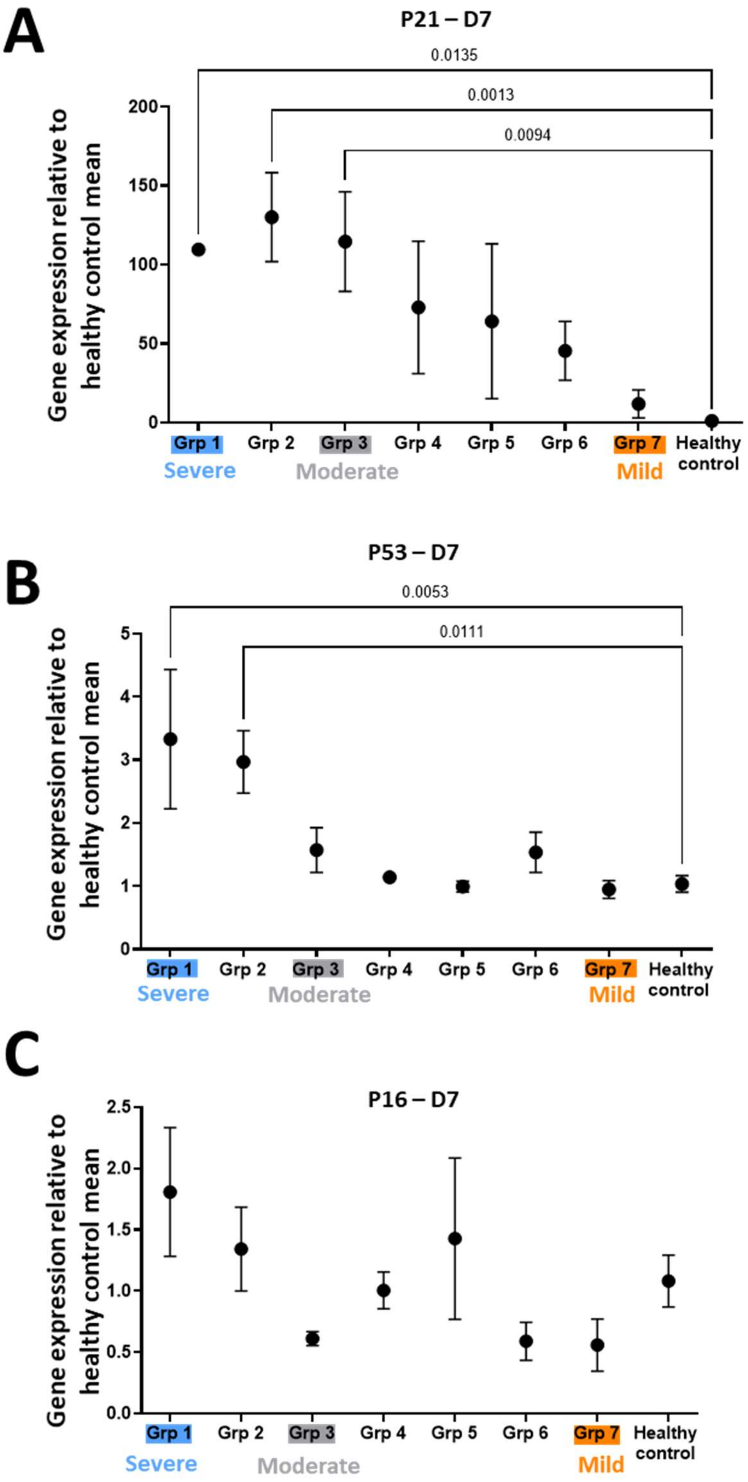

Supplementary Figure 4- Expression of senescence markers in whole liver tissue of Mdm2 mice administered a range of AAV8.TBG.Cre doses.

Expression of the senescence markers **a)** p21, **b)** p53 and **c)** p16 across the full senescence dose response analysed at D7 following AAV8.TBG.Cre induction. qPCR results were normalised to PPIA housekeeper and expressed as fold change relative to healthy control mean expression. N=3-5 mice per group analysed. Error bars are SEM. Ordinary One-Way ANOVA relative to healthy control mean with Dunnett's Multiple Comparisons test. Results show dose dependent increases in p21 and p53 expression of these senescent markers but no difference in p16 indicating that AAV8.TBG.Cre induces senescence along the p21/p53 axis.

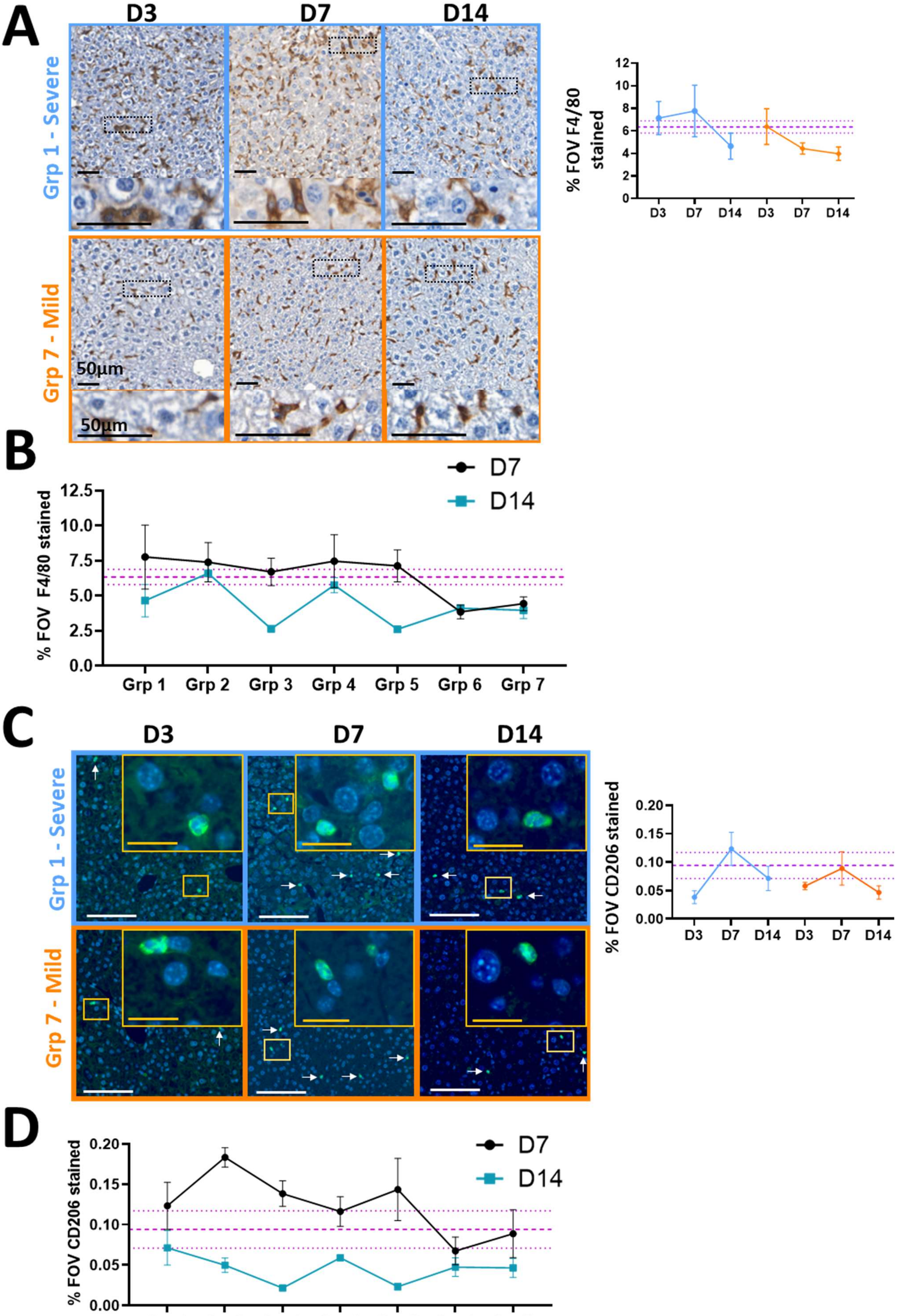
A
D3
D7
D14
Grp 1 - Severe
Grp 7 - Mild
50µm
50µm
% FOV F4/80 stained
12
10
8
6
4
2
0
D3
D7
D14
D3
D7
D14
B
D7
D14
% FOV F4/80 stained
12.5
10.0
7.5
5.0
2.5
0.0
Grp 1
Grp 2
Grp 3
Grp 4
Grp 5
Grp 6
Grp 7
C
D3
D7
D14
Grp 1 - Severe
Grp 7 - Mild
% FOV CD206 stained
0.20
0.15
0.10
0.05
0.00
D3
D7
D14
D3
D7
D14
D
D7
D14
% FOV CD206 stained
0.20
0.15
0.10
0.05
0.00
Grp 1
Grp 2
Grp 3
Grp 4
Grp 5
Grp 6
Grp 7

Supplementary Figure 5- Quantification results for tissue staining of macrophage markers after induction of different levels of senescence in Mdm2 mice.

Results of immunohistochemical tissue staining and quantification of Mdm2 mice administered with a range of doses of AAV8.TBG.Cre (group 1-7) to induce different levels of senescence. Quantification of staining based on mean±SEM % of the field of view (FOV) positively stained. N=3-5 independent animals per group and timepoint with ≥10 FOVs quantified per animal. Purple dashed lines are healthy control mean±SEM. **a)**Representative histological micrographs of the F4/80 pan macrophage marker in the Severe and Mild senescence injury dosing groups at D3, D7 and D14 post-induction with accompanying quantification. Scale bars 50µm. **b)** Quantification of F4/80 staining based on % of FOV at D7 and D14 for the entire dosing cohort. **c)** Representative micrographs of CD206 tissue staining in the Severe and Mild senescence injury dosing groups at D3, D7 and D14 post-induction with accompanying quantification. White arrows indicate positively stained cells. Insets are digitally magnified 1.5x. **d)** Quantification of CD206 staining based on % of FOV at D7 and D14 for the entire dosing cohort.

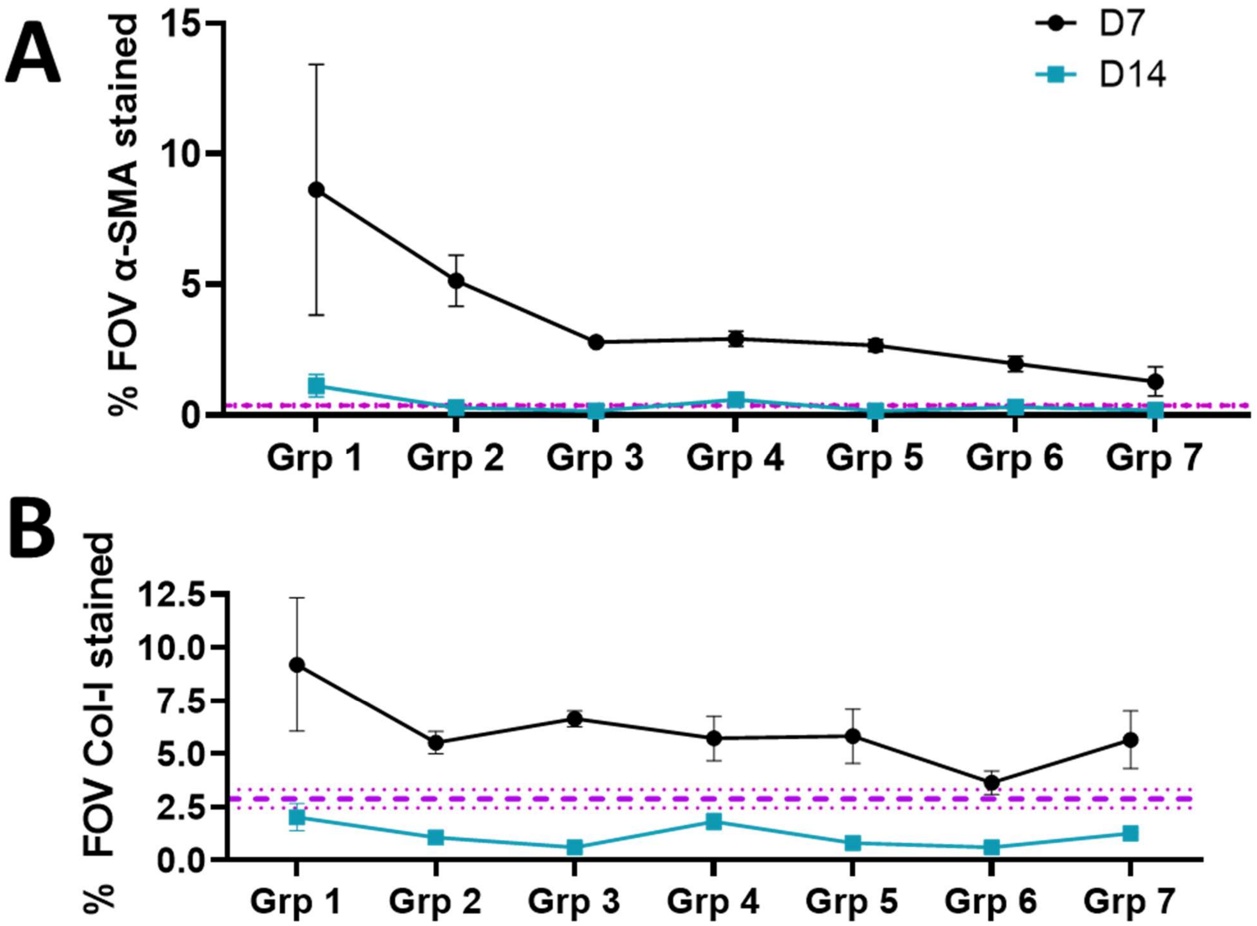

Supplementary Figure 6- Quantification results for tissue staining of tissue remodelling markers D7 and D14 after induction of different levels of senescence in Mdm2 mice.

Results of immunohistochemical tissue staining quantification of *Mdm2* mice administered with a range of doses of AAV8.TBG.Cre (group 1-7) to induce different levels of senescence at D7 and D14 **a)** α-SMA (activated myofibroblast marker) and **b)** Collagen-I (ECM marker). Purple dashed lines are healthy control mean±SEM. Data points represent mean N=3-5 independent animals per group and timepoint. All error bars are ±SEM.

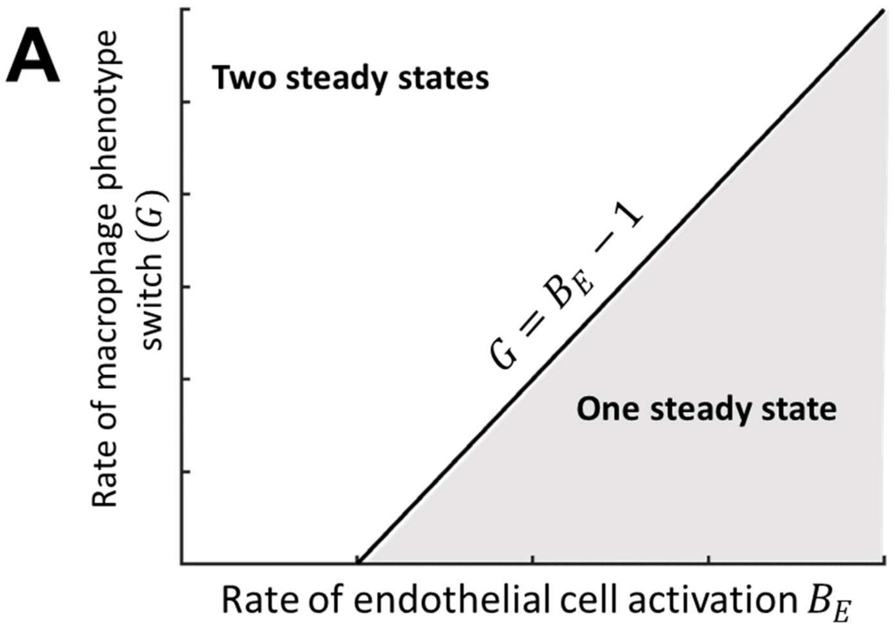

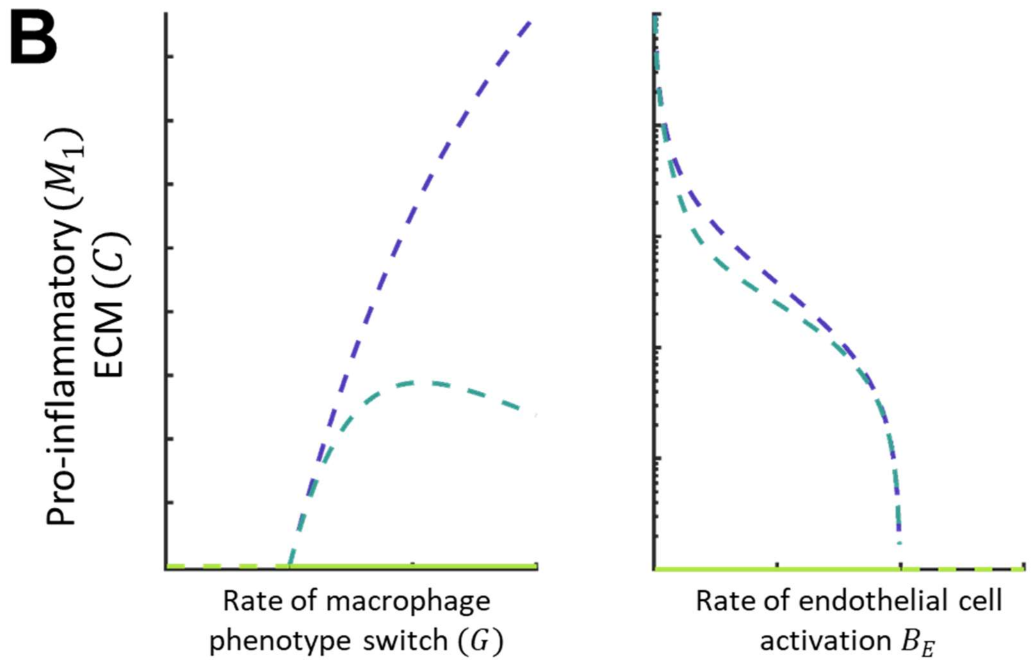

Supplementary Figure 7- Solution space for rate values and linear stability analysis in bifurcation graph

a) Solution space and number of steady states for different G and B_E.

b) Bifurcation diagrams for pro-inflammatory macrophages M_1 (purple line) and ECM C (light blue line) as a function of rate of macrophage phenotype switch G (with B_E set to a value different than 1 and the rest other parameters equal to 1) and rate of endothelial cells activation B_E (all parameters equal to 1). Solid and dashed lines represent stable and unstable solution branches respectively. The green line is the zero steady state. LHS: At a critical value of G, a second positive unstable steady branch appears. As G increases, C decreases approaching zero, however it always remains non-zero, positive and unstable. RHS: At a critical value of B_E, the two solution branches collapse into one, the zero one, which is unstable.

Supplementary Table 1- Primary antibody conditions used for immunostaining

Antigen	Supplier	Catalogue #	Species	Stock protein conc. (mg/mL)	Dilution	Antigen retrieval
CD206	Abcam	Ab64693	Rabbit	1	1/200	pre-warmed sodium citrate buffer (pH 6.0), 15 mins
F4/80	Abcam	Ab6640	Rat	1	1/100	15 minutes Proteinase K at 37°C
iNOS	BD	610329	Mouse	0.25	1/100	pre-warmed sodium citrate buffer (pH 6.0), 15 mins
α-SMA	Sigma	A2547	Mouse	2.0-6.0 (varies)	1/1000	5min pre-heat TE buffer (pH 9.0), 10 mins
Col-I	Southern Biotech	1310-01	Goat	0.2	1/300	5min pre-heat TE buffer (pH 9.0), 10 mins
ERG	Abcam	Ab92513<br>Ab214341	Rabbit<br>Mouse	0.88	1/125<br>1/500	DAB: pre-warmed sodium citrate buffer (pH 6.0), 10 mins<br>IF: TE 5min pre-warm + 10min
VCAM-1	Abcam	Ab134047	Rabbit	0.43	1/500	IF: TE 5min pre-warm + 10min

Supplementary Table 2- Details of primers used in qPCR analyses

Marker	Gene name	Quantitect #
Myofibroblast	ACTA2/α-SMA	QT00140119
Myofibroblast	PDGFRB	QT00113148
Inflammation	SOCS3	QT02488983
Tissue macrophage/Kupffer cells	CD68	QT00254051
Tissue macrophage/Kupffer cells	EMR1/F4/80	QT00099617
M2 macrophage	MRC1/CD206	QT00103012
M2 macrophage	CD163	QT00123074
M1 macrophage	CD86	QT01055250
M1 macrophage	CD80	QT00129787
Collagen ECM marker	Collagen 1A1	QT02589482
Collagen ECM marker	Collagen 1A2	QT02325736
Endothelial/endothelial proliferation	PECAM-1/CD31	QT01052044
Endothelial/endothelial proliferation	VEGFA	QT00160769
Endothelial/endothelial proliferation	ERG	QT01755551
Endothelial/endothelial proliferation	CDH5/VE-Cadherin	QT00110467
Endothelial/endothelial proliferation	FGA	QT00128198
Endothelial/endothelial proliferation	FGG	QT00101738
Endothelial/endothelial proliferation	VCAM-1	QT00128793
Acute endothelial injury/regeneration marker	ACKR3/CXCR7	QT00254443
Endothelial activation marker	ICAM-1	QT00155078
Senescence	CDKN2A/P16	QT00252595
Senescence	CDKN1A/P21	QT00137053
Senescence	TRP53/P53	QT00101906
Housekeeper	PPIA	QT00247709

Supplementary Note 1

Steady states

Steady state solutions (that have no time dependence) can be determined by setting the left hand side of the equations corresponding to time derivatives to zero (equations 1-6). We denote steady states variables with stars. We note that equations 1-6 have a zero steady state $(T^*, M_1^*, M_2^*, E^*, C^*, F^*) = (0, 0, 0, 0, 0, 0)$. To determine the non-zero steady states of the model, we set the right hand sides of equations 1-6 to zero and solve the resulting coupled algebraic equations. We reduce the system to two non-linear algebraic equations for F* and C* as follows:

$$F^* = \frac{B_F f(y^*)}{D + K_F g(y^*)}, C^* = \frac{B_C B_F f(y^*)}{[D + K_C g(y^*)]\,[D + K_F g(y^*)]}$$ Equation (8)

where

$$f(y^*) = \frac{D}{B_E} - \frac{y^*}{Dy^* + G}, g(y^*) = \frac{G}{D}\frac{f(y^*)}{y^*}, y^* = (1 + C^*)(1 + F^*)$$ Equation (9)

The steady state values of the remaining variables are then given by

$$T^* = 0, M_1^* = f(y^*), M_2^* = g(y^*), E^* = \frac{B_E f(y^*)}{D - B_E f(y^*)}$$ Equation (10)

We can use these results to determine conditions on the parameters to obtain the non-zero steady state. Since $C^*$and F^* are positive, we have that $y^* > 1$ and therefore

$f >= \frac{D}{B_E} - \frac{1}{D+G}$ and Equation (11)

$$g > \frac{G(D^2+DG - B_E)}{B_E(D^2+DG)}$$ Equation (12)

Since we also require $f > 0$ and $g > 0$ to ensure positive macrophage populations, we therefore find

$$G > \frac{B_E - D^2}{D}$$ Equation (13)

which is the condition on the parameters for two steady state solutions to co-exist.

Linear stability analysis

To determine the linear stability of the steady state solutions we introduce small amplitude perturbations of the form

$$T = \mathrm{T} + \varepsilon \hat{T} E^{\lambda t} + \mathrm{O}(\varepsilon^2)$$ Equation (14)

With similar expressions for M_1, M_2, E, C and F. Here λ is the growth rate of the perturbation, $\hat{T}$ etc are constants and $0 < \varepsilon \ll 1$ is a small parameter. Substituting the expressions 14 into equations 1-6 and retaining linear terms leads to an equation of the form

$\boldsymbol{J}x = \lambda x$ Equation (15)

where $\boldsymbol{J}(T^*, M_1^*, M_2^*, E^*, C^*, F^*)$ is the 6X6 Jacobian matrix given by

$$\begin{bmatrix} -K_T M_2^* - D & 0 & -K_T T^* & 0 & 0 & 0 \\ B_1 & -\frac{G}{(C^*+1)(F^*+1)} - D & 0 & 1 & \frac{G M_1^*}{(C^*+1)^2(F^*+1)} & \frac{G M_1^*}{(C^*+1)(F^*+1)^2} \\ B_2 & \frac{G}{(C^*+1)(F^*+1)} & -D & 0 & -\frac{G M_1^*}{(C^*+1)^2(F^*+1)} & -\frac{G M_1^*}{(C^*+1)(F^*+1)^2} \\ 0 & B_E(1+E^*) & 0 & B_E M_1^* - D & 0 & 0 \\ 0 & 0 & -K_C C^* & 0 & -K_C M_2^* - D & B_C \\ 0 & B_F & -K_F F^* & 0 & 0 & -K_F M_2^* - D \end{bmatrix}$$

Equation 15, $\boldsymbol{x}$ is the vector of unknowns $\boldsymbol{x} = (\hat{T}, \hat{M}_1, \hat{M}_2, \hat{E}, \hat{C}, \hat{F})$ and the eigenvalues $\lambda = \lambda_n \ (n = 1, \dots, 6)$ satisfy $|\boldsymbol{J} - \lambda \boldsymbol{I}| = 0$. Instability of a steady state is predicted when $\max_{\{n=1,\dots,6\}} R(\lambda_n) > 0$. Conversely, the steady state is said to be linear stable if $R(\lambda_n) < 0$ for $n = 1, \dots, 6$ where R is the real part of the eigenvalues.

The eigenvalues associated with the trivial steady state are real numbers. The largest eigenvalue is given by

$\lambda = \frac{1}{2}\left(-2D - G + \sqrt{4B_E + G^2}\right),$ Equation (16)

and we see that the stability of the trivial steady state depends only on G, the rate of phenotype switch and B_E, the rate of activated endothelial cell increase, and D. This eigenvalue is negative when

$B_E > 0, G > max\left\{0, \frac{B_E - D^2}{D}\right\}$ Equation (17)

which ensures the origin is stable point. This condition is also required for the existence of the non-zero steady state.

A similar linear stability of the non-trivial steady state indicates that it is always unstable to small amplitude perturbations (details not shown).

In **Supplementary Figure 7**, we present bifurcation diagrams showing how the steady-state solution structure varies as B_E and G are varied. All other parameters are set to 1. The transcritical bifurcation point corresponds to $G = B_E - 1$. In the left hand figure, as the parameter G decreases through the bifurcation point, the non-trivial steady state is lost and there is a change in stability for the trivial steady state from stable to unstable. A similar behaviour is observed in the right hand figure, as B_E increases through the bifurcation point.

Supplementary Note 2

Representative Fiji macro for automated quantification of stained regions

```
/* Macro for quantifying area of DAB staining in haemotoxylin and DAB stained tissues
 */

//CLEAR LOG
print("\\Clear");

// CLOSE ALL OPEN IMAGES
while (nImages>0) {
	selectImage(nImages);
   close();
}

//SET BATCH MODE
setBatchMode(false);   //true or false, true if you don't want to see the images, which is faster

//START MESSAGE
print("**** STARTING THE MACRO ****");

//INPUT/OUPUT folders
inDir=getDirectory("Choose the input folder");
outputDir=getDirectory("And the output folder");
myList=getFileList(inDir);  //an array

//Define your measurements and settings for
run("Set Measurements...", "area area_fraction limit redirect=None decimal=2");
roiManager("Set Line Width", 2);

//Defining arrays
ImageName=newArray();
PercentageSurfaceInDAB=newArray();
AreaInDAB=newArray();
ImageAnalysed=0;

for (j = 0 ; j < myList.length ; j++ ){
	path=inDir+myList[j];   //path to each file
	open(path);
	FileName=File.nameWithoutExtension;
	ImageID=File.name;
	Title=getTitle();
	print("Processing "+ImageID);
	ImageName=Array.concat(ImageName,Title);
	getStatistics(area, mean, min, max, std, histogram);


	//Thresholding the image to find DAB.  First split image by Colour Deconvolution and keep DAB 
channel
	selectWindow(Title);
	run("Set Scale...", "distance=3.9924 known=1 unit=µm global");
	run("Duplicate...", "title=DAB");
	run("Colour Deconvolution", "vectors=[H DAB]");
```

```
	selectWindow("DAB-(Colour_3)");
	close();
	selectWindow("DAB-(Colour_1)");
	close();
	selectWindow("Colour Deconvolution");
	close();
	selectWindow("DAB-(Colour_2)");

	//set threshold for DAB positive regions (modify depending on stain isotype control)
	run("Threshold...");
	setThreshold(0, 180);
	setOption("BlackBackground", true);
	run("Convert to Mask");

	//convert to binary image
	run("Measure");


	//updating area results
	AreaDAB=getResult("Area",j);
	AreaInDAB=Array.concat(AreaInDAB,AreaDAB);
	PercentAreaDAB=(AreaDAB/area)*100;
	PercentageSurfaceInDAB=Array.concat(PercentageSurfaceInDAB,PercentAreaDAB);


	//QC images with mask overlay
	selectWindow("DAB-(Colour_2)");
	run("Analyze Particles...", "size=0-Infinity add");
	selectWindow("DAB");
	roiManager("Show All without labels");
	RoiManager.setPosition(0);
	roiManager("Set Color", "yellow");
	roiManager("Set Line Width", 2);
	run("Flatten");

//saving QC image
	selectWindow("DAB-1");
	saveAs("Tiff", outputDir+Title+"_QC.tif");
	close();


	print("There was an area of "+AreaDAB+" stained, representing "+PercentAreaDAB+" percent of the 
FOV");

	close("*");

	roiManager("reset");
}

Array.show("Results", ImageName,AreaInDAB,PercentageSurfaceInDAB);
	saveAs("Results", outputDir+"Results.csv");
```

```
//saving log
print("***** Macro done *****");
selectWindow("Log");
saveAs("Text", outputDir+FileName+"_Log.txt");

close("*");
```

<u>Supplementary Note 3</u>

Representative Fiji macro for automated quantification of stained nuclei (ERG analysis)

```
/* Macro for quantifying area of DAB staining in haemotoxylin and DAB stained tissues
 */

//CLEAR LOG
print("\\Clear");

// CLOSE ALL OPEN IMAGES
while (nImages>0) {
		selectImage(nImages);
   close();
}

//SET BATCH MODE
setBatchMode(false);   //true or false, true if you don't want to see the images, which is faster

//START MESSAGE
print("**** STARTING THE MACRO ****");

//INPUT/OUPUT folders
inDir=getDirectory("Choose the input folder");
outputDir=getDirectory("And the output folder");
myList=getFileList(inDir);  //an array

//Define your measurements and settings for
run("Set Measurements...", "area area_fraction limit redirect=None decimal=2");
roiManager("Set Line Width", 2);

//Defining arrays
ImageName=newArray();
PercentageSurfaceInDAB=newArray();
AreaInDAB=newArray();
DABPatches=newArray();
ImageAnalysed=0;

for (j = 0 ; j < myList.length ; j++ ){
		path=inDir+myList[j];   //path to each file
		open(path);
		FileName=File.nameWithoutExtension;
		ImageID=File.name;
		Title=getTitle();
		print("Processing "+ImageID);
```

```
	ImageName=Array.concat(ImageName,Title);
	getStatistics(area, mean, min, max, std, histogram);


	//Thresholding the image to find DAB.  First split image by Colour Deconvolution and keep DAB
channel
	selectWindow(Title);
	run("RGB Color");
	run("Duplicate...", "title=DAB");
	run("Split Channels");
	selectWindow("DAB (green)");
	selectWindow("DAB (blue)");
	selectWindow("DAB (red)");
	close();
	selectWindow("DAB (green)");
	close();
	setAutoThreshold("Default dark");

	//run("Threshold...");
	setThreshold(150, 255);
	run("Invert");
	run("Analyze Particles...", "size=300-Infinity pixel add");
	DAB=roiManager("count");


	//Updating array to include the number of patches
	DABPatches=Array.concat(DABPatches,DAB);


	//Circumventing issues if there is no or only one DAB patch by creating single pixel ROIs
	if(DAB<=2){
		makeRectangle(0, 0, 1, 1);
		roiManager("Add");
		makeRectangle(1, 1, 1, 1);
		roiManager("Add");
		DAB=DAB+2;
	}

	selectWindow(Title+" (RGB)");
	roiManager("Show All without labels");
	roiManager("Combine");
	roiManager("Add");
	roiManager("Set Color", "yellow");
	roiManager("Set Line Width", 2);
	roiManager("Select All");
	roiManager("Measure");
	run("Flatten");



	//updating area results
	AreaDAB=getResult("Area",j);
	AreaInDAB=Array.concat(AreaInDAB,AreaDAB);
```

```
	PercentAreaDAB=(AreaDAB/area)*100;
	PercentageSurfaceInDAB=Array.concat(PercentageSurfaceInDAB,PercentAreaDAB);


	//QC images with mask overlay

	selectWindow(Title+" (RGB)-1");
	saveAs("Tiff", outputDir+Title+"_QC.tif");
	close();

	print("There were "+DAB+" ERG stained cells, with an area of "+AreaDAB+" stained, representing
"+PercentAreaDAB+" percent of the FOV");

	close("*");

	roiManager("reset");
}

Array.show("Results", ImageName,DABPatches,AreaInDAB,PercentageSurfaceInDAB);
	saveAs("Results", outputDir+"Results.csv");


//saving log
print("***** Macro done *****");
selectWindow("Log");
saveAs("Text", outputDir+FileName+"_Log.txt");

close("*");
```
